# Supplementary material for: Identification of VRK1 as a New Neuroblastoma Tumor Progression Marker Regulating Cell Proliferation
Source: Cancers (Basel). 2020 Nov 20;12(11):3465. doi: 10.3390/cancers12113465 (PMC7699843; doi:10.3390/cancers12113465)
Supplement: Supplementary file 1 [file cancers-12-03465-s001.zip › FigureS2+legend.pdf]

Figure S2

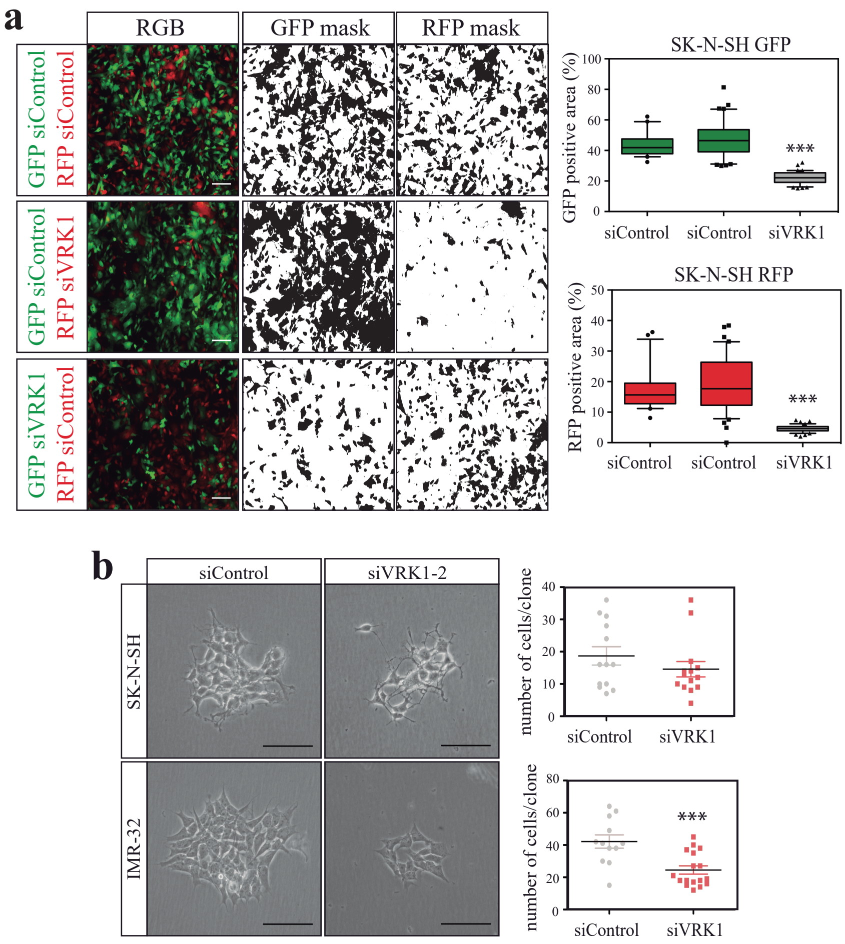

Figure S2. VRK1 downregulation abrogates proliferation. (**a**) Competition assay with SK-N-SH cells labelled either with GFP or RFP and in which only one of the populations was depleted of VRK1 with siRNA. Relative enrichment on cell population is shown; (**b**) Colony formation assay after siRNA transfection indicated that cell proliferation is impaired after VRK1 knockdown. Scale bars: 100 µm. *** p<0,001.
