# Supplementary material for: Identification of VRK1 as a New Neuroblastoma Tumor Progression Marker Regulating Cell Proliferation
Source: Cancers (Basel). 2020 Nov 20;12(11):3465. doi: 10.3390/cancers12113465 (PMC7699843; doi:10.3390/cancers12113465)
Supplement: Supplementary file 1 [file cancers-12-03465-s001.zip › FigureS3+legend.pdf]

Figure S3

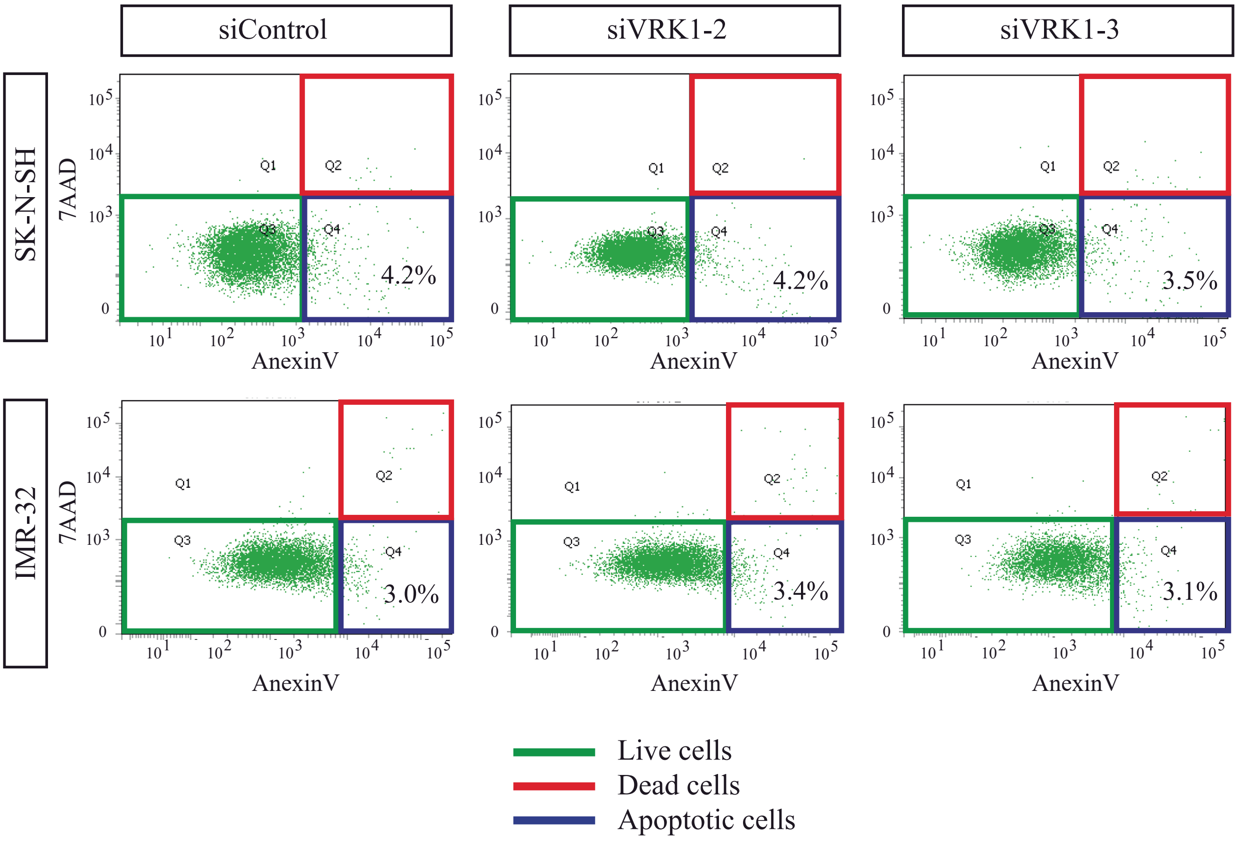

Figure S3. Apoptosis assay after VRK1 downregulation. Annexin V apoptosis assay on SK-N-SH or IMR-32 neuroblastoma cell lines treated with siRNAs for VRK1 or control. Percentage of apoptotic cells is shown.
