# Supplementary material for: Identification of VRK1 as a New Neuroblastoma Tumor Progression Marker Regulating Cell Proliferation
Source: Cancers (Basel). 2020 Nov 20;12(11):3465. doi: 10.3390/cancers12113465 (PMC7699843; doi:10.3390/cancers12113465)
Supplement: Supplementary file 1 [file cancers-12-03465-s001.zip › FigureS1+legend.pdf]

# Figure S1

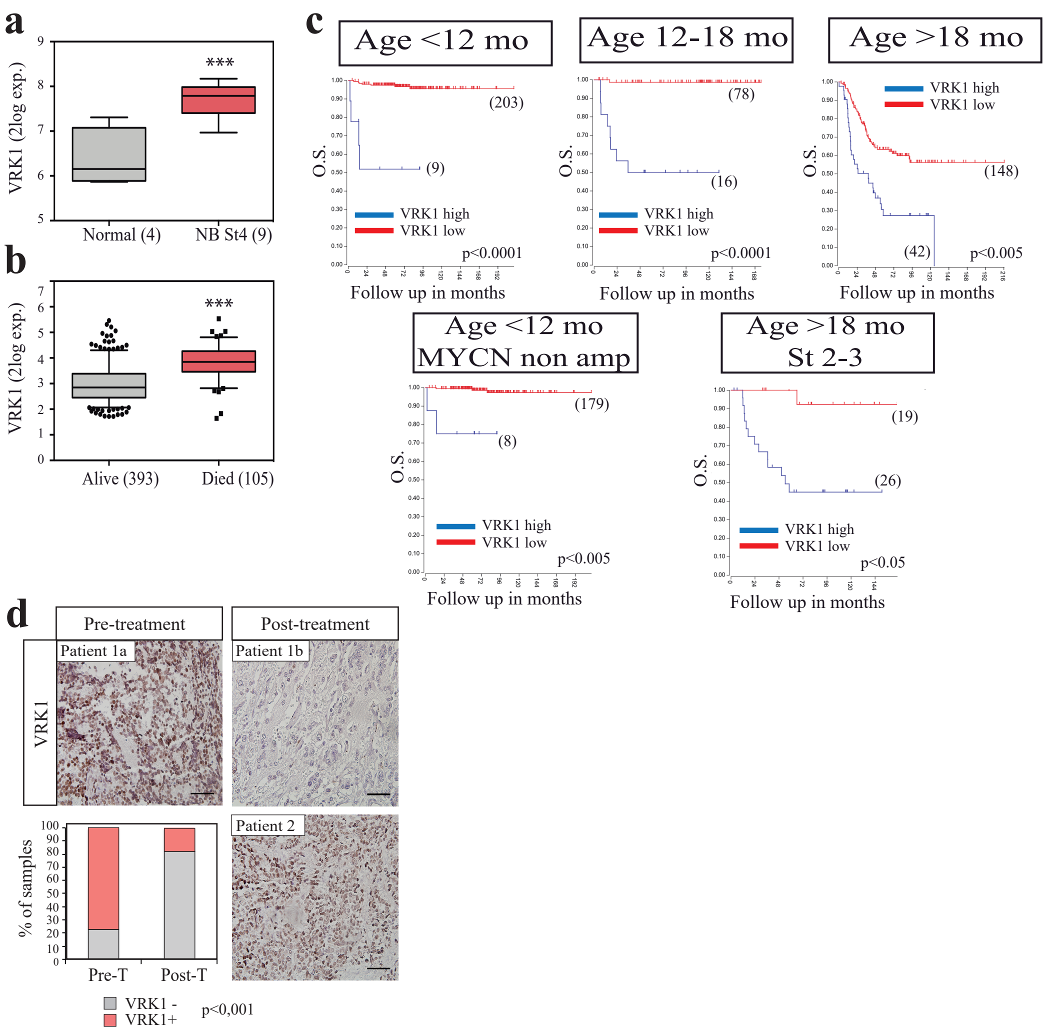


Figure S1. Expression of VRK1 in NB tumors. (a) Expression of VRK1 in stage 4 patient tumor samples compared with normal tissue (adrenal gland and fetal brain) (GSE54720); (b) VRK1 expression in patient tumor samples of all stages separated by outcome (GSE62564); (c)Overall survival probability Kaplan curves for patients on the indicated age at diagnosis group, startified according to VRK1 expression. O.S.:Overall survival, mo:months, St: INSS stage. (d) Immunohistochemistry showing VRK1 expression in a representative patient sample pre- and post-treatment (patient 1) and in a sample from a patient with relapse after treatment (patient 2). Quantification of VRK1 expression score in samples pre- or post-treatment is shown (13 and 22 samples respectively). Scale bar: 60 µm.
