# Supplementary figures and images for: Identification of VRK1 as a New Neuroblastoma Tumor Progression Marker Regulating Cell Proliferation
Source: Cancers (Basel). 2020 Nov 20;12(11):3465. doi: 10.3390/cancers12113465 (PMC7699843; doi:10.3390/cancers12113465)

Related to Figure 2

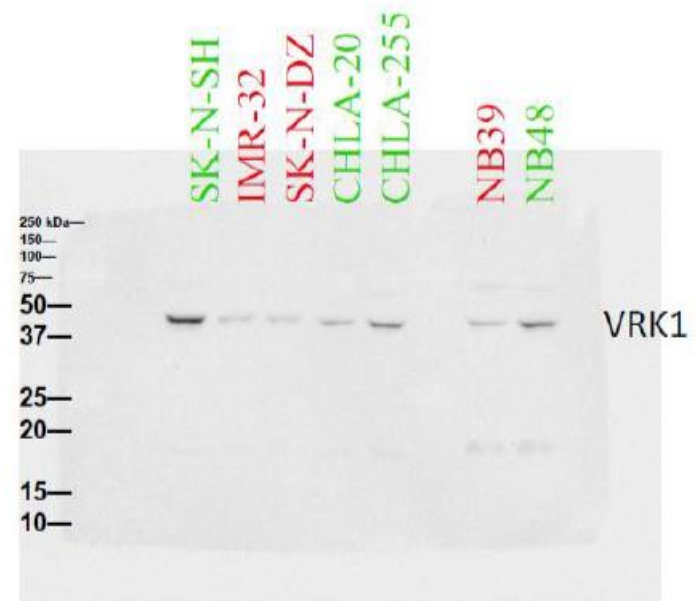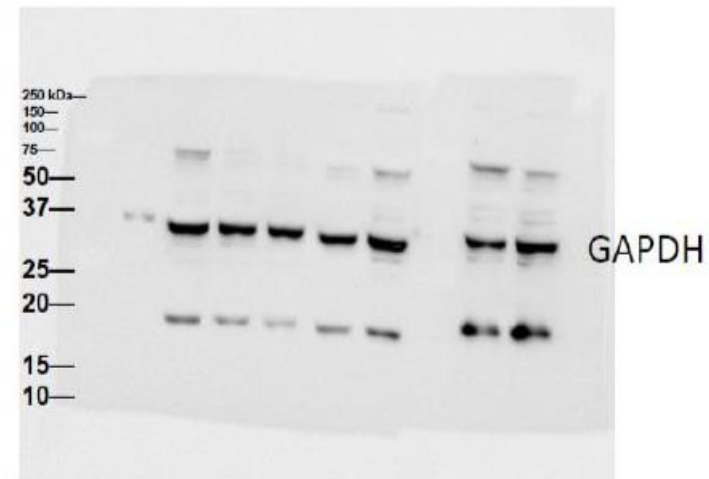

# SK-N-SH

## Complete Blots Figure 3

# IMR-32

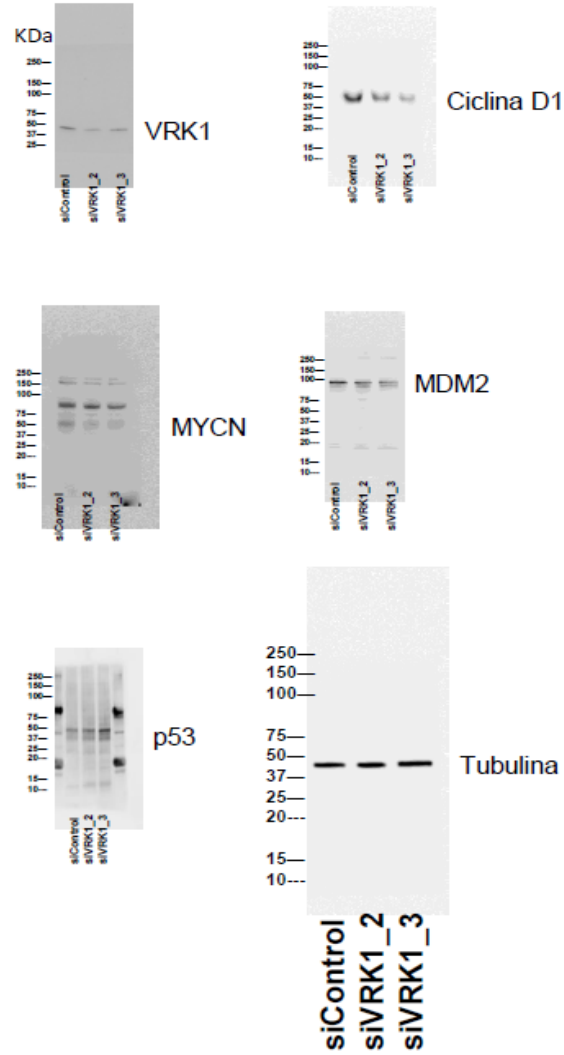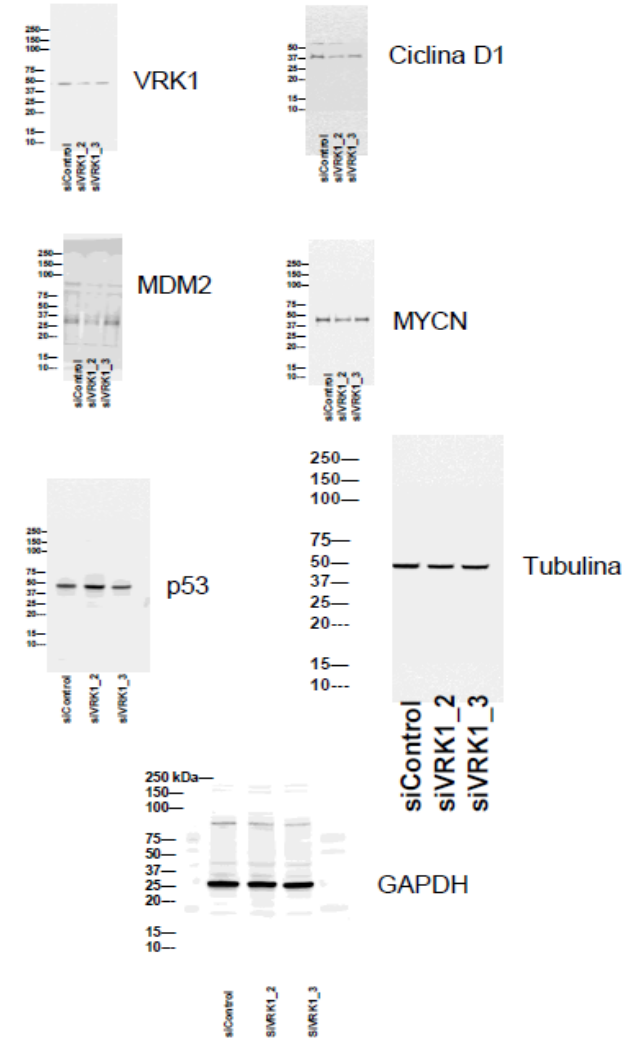

Supplement: Supplementary file 1 [file cancers-12-03465-s001.zip › Figure S4.pdf]
